# Lung cancer in never smokers (LCINS): development of a UK national research strategy

**DOI:** 10.1038/s44276-023-00006-w

**Published:** 2023-07-20

**Authors:** Sam Khan, Nathaniel Hatton, Daniel Tough, Robert C. Rintoul, Coral Pepper, Lynn Calman, Fiona McDonald, Clare Harris, Amelia Randle, Michelle C. Turner, Ruth A. Haley, Janette Rawlinson, Philip A. J. Crosbie, Frank McCaughan, Matthew Hatton

**Affiliations:** 1 Leicester Cancer Research Centre, University of Leicester, Leicester, UK; 2 Division of Oncology, Leeds Teaching Hospital Trust, Leeds, UK; 3 Department of Education, Health and Lifelong Learning, Bishop Grosseteste University, Lincoln, UK; 4 Department of Oncology, Royal Papworth Hospital, University of Cambridge, Cambridge, UK; 5 Library and Information Services, University Hospitals of Leicester NHS Trust, Leicester, UK; 6 Centre for Psychosocial Research in Cancer, School of Health Sciences, University of Southampton, Southampton, UK; 7 Department of Oncology, Royal Marsden’s Hospital, London, UK; 8 Department of Medicine, University of Cambridge, Cambridge, UK; 9 Somerset, Wiltshire, Avon and Gloucestershire Cancer alliance, Cambridge, UK; 10 Barcelona Institute for Global Health (ISGlobal), Barcelona, Spain; 11 Formerly National Cancer Research Institute (NCRI), Madrid, Spain; 12 Patient representative, Sandwell, UK; 13 Division of Infection, Immunity and Respiratory Medicine, University of Manchester, Manchester, UK; 14 Heart and Lung Research Institute, Department of Medicine, University of Cambridge, Cambridge, UK; 15 Weston Park Hospital, Sheffield Teaching Hospital Trust, Sheffield, UK

## Abstract

**Introduction:**

Lung cancer in never smokers (LCINS) accounts for 15% of lung cancers diagnosed in the UK, making it the 8th most common cancer. There are few robust studies specific to the LCINS population making data surrounding the incidence and mortality of LCINS incomplete, leaving many gaps in our understanding of the needs of this population.

**Methods:**

To address a lack of research in this important area, the UK National Cancer Research Institute Lung Study Group (NCRI-LSG) undertook a national survey and hosted a research strategy day to define key research priorities. A wide cross section of stakeholders, including patient advocates, the charitable sector, basic and translational researchers, and multi-disciplinary healthcare professionals contributed highlighting their research priorities.

**Results:**

One-hundred twenty-seven surveys were completed (52 by patients/patient advocates) prior to the strategy day. These identified themes for expert review presentations and subsequent workshop discussions at the national research strategy day, which registered 190 attendees (50 patients/patient advocates). The four key themes that emerged to form the basis of a research strategy for LCINS are (1) Raising awareness, (2) Risk assessment and early detection, (3) Disease biology, (4) Living with and beyond.

**Conclusion:**

This paper summarises current evidence and important gaps in our knowledge related to LCINS. We present recommendations for a national research strategy aimed at improving outcomes for patients.

## Introduction

Lung cancer is the world’s leading cause of cancer-related death and it is estimated that 10–25% of all diagnoses are made in individuals who have never smoked [1]. Lung cancer in never smokers (LCINS) is the 7th leading cause of cancer death for men and women worldwide [2]. The widely accepted definition of a ‘never-smoker’ (NS) is an individual who has smoked less than 100 cigarettes in their lifetime [3]. In the UK, LCINS is responsible for an estimated 6000 deaths per year making LCINS the 8th most common cancer in the UK if considered as a distinct disease entity [4]. The demographic of LCINS differs significantly from lung cancer occurring in the smoking population; the majority of cases occurring in women [5], presenting at a younger age [6, 7] commonly adenocarcinomas [3] with significantly higher frequencies of driver mutations [8]. For the majority of LCINS symptoms are non-specific [9], often dismissed by patients and primary healthcare providers, meaning patients have advanced disease at presentation [10] with a significant proportion diagnosed at emergency presentation [11]. Although, survival for LCINS appears better than for the smoking population in prospective studies, it remains disappointingly low [12].

There are few robust studies specific to the LCINS population making data surrounding the incidence and mortality of LCINS incomplete, leaving many gaps in our understanding of the needs of this population. A review of the UK Lung Cancer trial portfolio (January 2023) showed that of 150 ‘open’ studies none are exclusively focused on the LCINS population. Having identified the paucity of specific research focused on LCINS the Steering Committee of the National Cancer Research Institute’s Lung Group (NCRI-LG) recognised that specific needs of this population are likely to be different to the smoking lung cancer populations and set out to understand those needs though a survey and research strategy day. Recognising that they may be specific needs at all stages of the patient journey we elected to take an open approach using responses to the on-line survey taking specific needs highlighted by multiple responders forward for a more detailed review on the strategy day. We have structured this report around 4 key themes identified through this process.

## Methods

The NCRI-LG undertook a survey and a one-day meeting to review and identify evidence gaps for the diagnosis and management of LCINS. The potential for evidence gaps to exist across the patient journey from diagnosis through to survivorship was recognised. An open approach was taken with the survey used to prioritise potential research questions for workshops within a meeting to formulate the research strategy for the NCRI-LG, which will be developed into workstreams to inform and design future studies focusing on improving outcomes for patients.

A bespoke survey was designed to collate ideas for research questions (Appendix A). The survey contained 6 categories (basic science, prevention, screening/early detection, diagnosis, treatment and survivorship). Within each of those categories, respondents were asked to list their top 5 research questions. The outcomes of the survey were grouped to formulate the workshop agenda (Appendix B) and discussion topics for breakout sessions (Appendix C).

Attendance was open to the UK lung cancer community and invitations were circulated through the multi-disciplinary professional bodies (including NCRI newsletters and study groups, the British Thoracic Oncology Group (BTOG)), patient organisations (NCRI patient forum, NCRI partner charities and UK lung cancer-specific charities) and social media. Those registering interest completed a survey to highlight their research priorities prior to the meeting. The results were used to identify themes for the workshops to explore in greater detail. The meeting was held virtually in May 2021. Participants (Table 1) were assigned to two workshops, Early Detection or Treatment, with discussion moderated to cover discovery/biology, Living with and Beyond Cancer (LWBC) and clinical research focusing on patient outcomes. The discussions were recorded, transcribed and collated with data from the survey by members of the NCRI secretariat to produce themes centred around the unmet needs of the LCINS population and research priorities. A further survey came after the strategy day, for individuals to choose their top three research questions. An average ranking of results was used to obtain the order of priorities. The themes are presented with background information and recommendations for research.

**Table 1:** Outline of registered delegates (*N* = 190) and breakdown of those completing survey (*N* = 127).

Breakdown of delegates	Number of participants
Total registered—strategy day	190
Total—completed survey	127

Breakdown of those completing survey	Number of participants
Patient advocates	52
Clinicians/consultants or academics	27
Allied health professionals	15
Scientists	14
Students or early career researchers	7
Government, charity or research managers	5
Industry	4
Unknown	3

## Theme 1: Raising awareness

### Patient and public comments

Raising awareness of LCINS, reducing barriers and delays to diagnosis were repeatedly highlighted from the surveys as an area which should be prioritised—‘*Much more awareness is needed both in the medical profession and general public*’. Attendees believed that more is needed to reduce the stigma surrounding lung cancer—‘*the respiratory doctor asked if I was a heavy smoker but I am a fifty year old never smoker*’, whilst highlighting the symptoms of the disease to aid quicker diagnoses—

*‘It needs to be stressed that vague symptoms can be cancer’. ‘Many of the (LCINS) patients I’ve met, like me had some sort of history of repeated chest infections yet were sent away with inhalers, antibiotics, steroids over a considerable time—many more had treated themselves for their non-specific symptoms by over-the-counter remedies bought in supermarkets or pharmacies. Several were told by their GP that their symptoms may be menopause.’*

### Evidence

The earlier detection of symptomatic cancer in general is associated with improved patient outcomes although there are no specific data available for individuals with LCINS [13]. 70% of patients who are diagnosed with cancer first report symptoms to Primary Care [14]. There are several factors that contribute to the delayed presentation of symptomatic lung cancer related to primary care/the healthcare system, the patient and the disease [15, 16]. Symptoms commonly associated with lung cancer, such as cough and breathlessness, are non-specific [17]. This can make it challenging for healthcare professionals to identify those patients requiring immediate investigation [18], which can lead to diagnostic delay [19]. In one study, a quarter of lung cancer diagnoses involved an avoidable delay and one third required three or more consultations with their GP prior to specialist referral [20, 21]. A higher number of pre-referral consultations is associated with a more negative experience of cancer care [22].

#### Clinical presentation

Little research has examined the clinical presentation of LCINS [23]. Retrospective series suggest symptoms are similar irrespective of smoking status, although haemoptysis may be less frequent in never smokers [9, 24]. There is some evidence that never smokers have more advanced disease at diagnosis [3, 24, 25] but this is not a consistent finding [26]. The widely known link between smoking and lung cancer may inadvertently lead patients who have never smoked and healthcare professionals to attribute symptoms to other causes [27]. In one study, never smokers took longer to seek medical attention compared to ever smokers (3 versus 2 months) [24] though this finding was not replicated in another study [28]. Despite this never smokers with lung cancer are reported to have better survival than smokers [12, 25, 26, 29–31]. The anecdotal nature of evidence around the clinical presentation of lung cancer in never smokers, where first symptoms can be a small change in ‘sporting’ performance, represents a very important gap in our knowledge.

#### LCINS awareness

Raising public awareness of LCINS through a media campaign is one approach that could reduce the delay in patients reporting symptoms. The potential benefit of such an approach was demonstrated by an increase in early lung cancer detection following a national awareness campaign highlighting persistent cough (3 weeks or longer) as a possible symptom of lung cancer. A public and healthcare professional lung cancer symptom awareness campaign in Leeds increased chest X-ray referral rates and the detection of early-stage disease [32]. A study in Doncaster combining a community-based social marketing intervention with GP education also had a positive impact on lung cancer detection [33]. It is unknown whether an education/awareness campaign specifically focused on LCINS would have an equivalent impact [34].

### Recommendations

- Develop interventions to raise awareness of LCINS at local, regional and national level. Interventions should include educational resources to improve knowledge about LCINS for healthcare professionals, in an accessible format for the public. There should be explicit information that anyone can get Lung Cancer and prior good health, diet and exercise capacity does not preclude the diagnosis.
- Interventions and educational resources used to improve awareness should be co-designed by key stakeholders including those with lived experience of LCINS. To help prompt referral and investigations for an alternative diagnosis if the clinical presentation is atypical.
- Interventions should be designed to ensure equity of access to information irrespective of age, sex, race, digital access and geographical locality. In practice this will require innovation in communication with the general population
- Further research is needed to explore the patient journey from development of symptoms to diagnosis of LCINS to identify factors/barriers to earlier presentation.

## Theme 2: Risk assessment and early detection

### Patient and public comments

A critical question raised by patients is ‘why me?’, given the much-emphasised direct association between smoking and lung cancer and the stigma associated with the disease. A clearer understanding of the risk factors for LCINS would have a major impact on raising awareness, avoiding risk and the development of screening protocols for the early detection of LCINS in the UK. Participants suggested the implementation of routine screening following identification of risk factors of LCINS to detect the disease when at its most treatable, improving survival rates—

ʻIs it possible to produce a health app which prompts a number of regular screening questions, which would advise seeking appropriate medical advice?ʼ.

To achieve this, we need research programmes that comprehensively assess germline (inherited) risk, the impact of respiratory co-morbidities, environmental and occupational exposures, and the molecular pathobiology of LCINS. Given the difficulties in identifying these risk factors, one participant questioned the feasibility of screening for lung cancer in never smokers, suggesting the focus should be upon education—‘*Can we realistically even consider screening for lung cancer in never smokers, should the focus be on education and awareness, so that concerns about lung cancer symptoms in never-smokers are not so frequently dismissed…*’.

This highlights the disparity between current knowledge and an idealistic early screening assessment.

### Evidence

#### Risk factors

##### Environmental exposures

Several environmental exposures are associated with increased risk of developing LCINS. Such exposures may be related to a person’s location, occupation, methods of cooking or heating and exposure to environmental tobacco smoke (passive smoking) [35, 36]. Living in an area with high background levels of radon is associated with an increased risk of developing lung cancer [37–39]. There is strong evidence that pollution, specifically particulate matter in outdoor air pollution, is carcinogenic in humans [40, 41] accounting for some of the global lung cancer incidence [42]. Higher ambient particulate matter (PM_2.5_) exposures were associated with higher lung cancer mortality in never smokers [43]. Other lung cancer risk factors include indoor emissions from household coal combustion, asbestos, silica dust, arsenic, welding fumes, diesel engine exhaust, various metals and other occupational processes [44–46]. Recently opium consumption was classified as carcinogenic for lung cancer [47].

Several gaps in our knowledge remain including interactions of environmental and occupational agents, identification of vulnerable exposure time windows or subpopulations, or in the case of complex exposures, identification of the most relevant or responsible agent [45, 48–50]. Occupational agents have typically been understudied in women [51]. There is a range of other environmental and occupational agents where the strength of the evidence in humans remains limited [52]. Little is known regarding the impact of exposure to environmental and occupational agents on lung cancer survival post diagnosis. Information on the effectiveness of specific interventions for cancer prevention is also often scarce.

##### Genetic susceptibility

LCINS is more common in females and in some ethnic groups [53, 54]. The female preponderance is poorly understood but is maintained across different ethnicities and national cohorts from South-East Asia to the US. In a large prospective study of women in the UK, risk factors for the development of LCINS included non-white ethnicity, asthma requiring treatment and taller stature [55]. A potential role for oestrogen has been mooted but the evidence is mixed, the prospective UK study did not show any link to the age of menopause or post-menopausal use of hormonal therapies [55]. In general, epithelial malignancies increase with age but LCINS is typically associated with a younger demographic than that of the smoking lung cancer population [24, 56–58].

The genetic predisposition to lung cancer has been well studied and multiple genetic loci/single-nucleotide polymorphisms associated with lung cancer risk [59]. More recently studies have examined germline risk in LCINS, particularly in Asian cohorts. There are both shared loci between smokers and never-smokers and distinct loci that segregate with LCINS or ever-smoker lung cancer. It is not clear how these findings translate to a UK population. A study in a Chinese population demonstrated how a polygenic risk score developed using SNPs could stratify individuals (including never smokers) according to lung cancer risk [60]. One interesting germline risk is the inherited SNP associated with the T970M mutation in EGFR, a mutation, which confers resistance to EGFR-specific tyrosine kinase inhibitors. This risk allele has been reported in US and in Asian cohorts [61–65]. There is an obvious need to understand the potential contribution of germline susceptibility in a UK population.

##### Other factors

The consumption of meats, alcohol, and a low fruit and vegetable intake has been suggested as a potential risk factor for lung cancer, although findings are contradictory [66, 67]. There are well-established links between lung cancer and other lung diseases, notably Chronic Obstructive Pulmonary Disease (COPD) and idiopathic pulmonary fibrosis. There is also an excess risk of lung cancer in other inflammatory lung diseases, including prior tuberculosis, bronchiectasis and asthma. However, individuals can have multiple diagnostic labels e.g. COPD and bronchiectasis and the relative contribution of the lung disease compared to smoking and environmental exposure is poorly studied. Prior radiotherapy (RT) is yet another risk for LCINS, and it is evident that even regimes where lower volumes of lung are treated, eg adjuvant breast cancer, are associated with an excess risk of lung cancer.

#### Lung cancer screening

Randomised controlled trials provide conclusive evidence that screening higher risk smokers with low-dose computed tomography (LDCT) scans reduces lung cancer-specific mortality [68, 69]. These studies used age and a threshold of smoking exposure (e.g., ≥30 packyears) to select the screening population. However, the more precise targeting of screening, based on individually calculated lung cancer risk scores, may improve screening performance [70, 71]. In the UK, the Targeted Lung Health Check (TLHC) programme has assessed the feasibility of LDCT screening implementation and led to the UK National Screening Committee formally recommending national adoption of targeted screening for lung cancer. Screening eligibility for this targeted programme is based on age (55–74 years) and individual risk score calculated using two risk prediction models PLCO_M2012_ [72] and LLP_v2_ [73]. Irrespective of other risk factors never smokers are not eligible for this programme.

At present there are no CT screening studies focussed on never smokers in Europe or North America. In Japan, one lung cancer screening programme demonstrated similar lung cancer detection in never smokers compared to smokers with less than 30 packyears smoking exposure [74]. In Taiwan, opportunistic LDCT screening has been promoted for several years. This has resulted in a 6-fold increase in early-stage disease detection in women (95% of whom are never smokers) and doubling of 5-year survival. However, there was no reduction in advanced lung cancer suggesting this approach is driving over-diagnosis rather than detection of clinically meaningful cancers [75]. The Taiwan Lung Cancer Screening for Never Smoker Trial (TALENT) is evaluating a targeted approach for LDCT screening in never smokers. Inclusion criteria included never smoking, age (55–75 years) and one of—family history of lung cancer (1st-3rd degree), passive smoking exposure, TB/COPD and high cooking index. A total of 12,011 individuals had a baseline LDCT scan. The prevalence of invasive lung cancer at baseline was 2.6% and 1.6% in those with and without a family history of lung cancer [76].

Modelling studies show that it is very challenging to identify never smokers at high risk [77] and previous reviews concluded that never smokers should not be screened as the benefits do not outweigh the potential risks such as radiation, over-diagnosis, unnecessary biopsies and treatment [78]. Therefore, novel approaches, driven by enrichment based on other risk factors or biomarkers are required.

### Recommendation

- Establish a UK registry of patients with LCINS. This should leverage the existing national infrastructure within the National Health Service (NHS) for recording lung cancer cases, linked with other resources such as the National Lung Cancer Audit and Clinical Practice Research Datalink (CPRD), which may facilitate ‘high-level’ identification of risk factors.
- Establish a prospective cohort of patients diagnosed with LCINS to collect detailed demographics, medical/occupational history and risk factors. This cohort would be given the opportunity to consent for future in-depth surveys to establish individual environmental and occupational risks and co-morbidities associated with LCINS. It would generate data representing the heterogeneous populations affected by LCINS including potentially under-represented groups (ethnicity, social deprivation). It would directly address the paucity of published large-scale data regarding UK LCINS populations.

The NHS/UK infrastructure is well suited to this approach. The successful development of a clinically and exposure annotated registry with paired archived samples and blood would allow molecular markers of germline risk and somatic mutational events to be related directly to exposure to putative environmental carcinogen (Theme 3). The objective would be to develop a risk score (based on demographics, exposure and molecular biomarkers) with a view to future stratification of a population for screening.

## Theme 3: Disease biology

### Patient and public comments

The importance of further research to understand underlying biological mechanisms that play a role in the development of LCINS was highlighted—

ʻ…even if you move away from radon and live in a bubble of pure air, perfect diet, perfect fitness you may have a genetic mutation no one knows about.ʼ

This might help address the ‘*why me?*’ question that often arises when a diagnosis of LCINS is made. ‘*The majority of the population is unaware that 1 in 2 of us will develop a cancer in our lifetime and then to develop a cancer that has only ever been associated with a lifestyle habit they’ve not had adds to their anger, fear and anxiety*’.

A deeper understanding of the pathobiology of LCINS could lead to strategies to prevent lung cancer and the development of more effective treatments. Identification of markers that identify aggressive disease or tumours with rapid tumour progression was mentioned in several questionnaires and how biomarkers might be used to promote earlier detection—

ʻmight molecular genetic biomarkers allow us to identify those at higher risk of lung cancer, for inclusion in cancer screening?ʼ.

### Evidence

A better understanding of the pathophysiology of LCINS has the potential to impact on the early detection and improved management of the disease. Most of the large-scale multi-omics studies on lung cancer have focused on the more common smoking-associated lung cancer in ever smokers. More recently, key studies have interrogated LCINS, drawing attention to the marked molecular differences between LCINS and ever smokers [79–82]. These studies have focused on adenocarcinoma, the dominant pathological subtype [83–86].

Broadly speaking three key observations have been made. The first is that the mutational signatures in LCINS are distinct from lung cancer in ever smokers (LCIES). The second is that the profile of mutational events affecting ‘driver’ oncogenes is different in LCINS and often involves druggable mutations. Finally, the genomic characteristics; tumour mutational burden (TMB) and aneuploidy are distinct in LCINS with a tendency towards a significantly less disrupted genome and a lower TMB in LCINS [79].

#### Mutational signatures in LCINS

Tobacco-smoking is associated with specific mutational signature, for example single-base substitution (SBS4) C > A/G > T. This was not detected in LCINS in one large survey and was infrequent in another [79, 87]. The important implication is that passive cigarette-smoking is not a dominant cause of LCINS. The data suggest some potential alternative endogenous and exogenous mechanisms.

Apolipoprotein B mRNA editing enzyme catalytic polypeptides (APOBECs) are a family of DNA-altering enzymes (cytosine deaminases) that target-specific sequence couplets for mutagenesis. In regions of East Asia (residents of Taiwan) where EGFR-mutated LCINS is common there is a suggestion that LCINS is associated with an APOBEC signature in younger females but has more of an exogenous mutational profile, including nitrated polycyclic hydrocarbons (perhaps from diesel exposure) in older females, observed in primarily stage 1 cancers [79]. In the Sherlock-Lung Study involving patients from the United States of European ancestry and those harbouring Stage 1–3 disease, a signature associated with reactive oxygen species was reported in 46% of cases, suggesting that oxidative stress may be a key genotoxic carcinogenic mechanism in LCINS [79].

#### Mutational hot-spots in LCINS

There are multiple mutational events regarded as key ‘drivers’ of lung carcinogenesis that are markedly enriched in LCINS compared to LCIES. The most common are EGFR mutations; others include ALK and ROS translocations and more rarely mutations in KRAS (G12C), BRAF, MET exon 14 skipping, ERBB2, AKT1 and NTRK [88, 89]. Some studies have attempted to describe potential links between certain occupational exposures and driver mutations [90]. The treatment options are well reviewed [89] but the key message is that many LCINS have potentially actionable mutations [88]. We therefore recommend that comprehensive targeted sequencing of biopsies or alternatives e.g. ctDNA in LCINS is mandatory to guide treatment.

Somatic mutational signatures in both LCINS and LCIES vary with ethnicity and gender. LCINS has been particularly associated with South-East Asian ancestry, as discussed above. Importantly, recent evidence from a Latin American population showed that Native American ancestry is more important than environmental triggers in contributing to somatic mutational profiles [91]. Furthermore, ancestry can impact on the interpretation of tumour mutational burden and the efficacy of immuno-therapeutics [92].

More pertinent to LCINS in the UK population, Pirie et al reported an increased relative risk (RR) of LCINS in non-white compared to white women (RR  =  2.34, 95% CI 1.55–3.52, *p*  <  0.001) [55]. The predominance of LCINS in women has raised the possibility of a hormonal role in pathogenesis, although a mechanistic understanding remains elusive [93, 94].

Given the significant impact of gender and ancestry on LCINS incidence, somatic profiles and even potentially therapeutic response, there is a clear knowledge gap regarding the demographics and somatic profile of LCINS in the UK. Closing this gap through a targeted LCINS registry will aid the prevention of burgeoning health inequalities [81].

#### Genomic characteristics of LCINS

Cancers are characterised by disrupted genomes but there are significant differences between LCINS and LCIES. TMB is a biomarker that, with reservations, is associated with smoking and with predicted neoantigen burden and response to immunotherapy (IO) agents [80, 95, 96]. In the Sherlock-Lung study which reported on 232 LCINS patients [79], fresh-frozen tumour specimens were analysed using high-coverage whole genome sequencing. The TMB in LCINS was less than 1/7th of an LCIES cohort. Nevertheless, it is critical to acknowledge and understand the heterogeneity within LCINS and the implications for therapeutic efficacy [97]. The Sherlock group suggested 3 classifications of LCINS tumours; piano, forte and mezzo-forte based on differences in the cancer genome structure. Piano subtypes demonstrated a low mutational burden with infrequent whole genome doubling, a forte subtype with predominant whole genome duplication and mezzo-forte subtype enriched [79, 97] for chromosomal arm-level amplifications. HLA disruption was relatively rare, reported in only 5.7% of cases. Notably, those with mutated TP53 have elevated TMB and have been associated with reduced survival [98].

As well as TMB, cancer genomes typically exhibit structural variants such as whole genome doubling, somatic copy-number aberrations (SCNAs) and aneuploidy. However, in LCINS a significant proportion have a very ‘quiet’ genome with no detectable SCNAs (piano subtype) [79, 97]. This is consistent with the TMB data and reflects a fundamental difference between LCINS and LCIES.

The implications for precision oncology are significant. The biomarkers used to guide IO choices in LCIES are imperfect. Given IO is typically less effective in LCINS, further studies are needed to understand the mechanisms involved and to define those LCINS cases who may benefit from IO. For example, one suggestion from a Taiwanese cohort [80] was that APOBEC mutational signature status may predict with IO sensitivity in an EGFR-WT subgroup. The authors acknowledge the impact of germline/ancestry as well as country-specific environmental exposures on LCINS, reinforcing the need for a UK-specific approach.

### Recommendation

- Establish a multicentre or UK patient registry/biobank for LCINS. This would describe the characteristics of the at-risk population in the UK. It would mandate the collection of archived samples, or for centres with appropriate infrastructure, their prospective and longitudinal collection. The registry would be associated with a questionnaire to define demographics and environmental exposure (Theme 2). The intention would be to perform a deep-dive professional interview with as many patients with LCINS as possible to fully understand their potential exposure history.

As noted above this would have far-reaching impact. It would facilitate a range of studies including an in-depth analysis (pan-‘omics) of the molecular epidemiology of UK LCINS and the interaction between environmental exposure, germline risk, interaction with microbiome and somatic events in LCINS. This dataset would inform whether there is a future potential for screening for LCINS in the UK a key aims of our patient population. It would provide an evidence base for experimental medicine approaches modelling and interplay between germline and environmental risks and directly inform novel target identification or target prioritisation for preclinical evaluation and therapeutic development for treatment or prevention. This would be a resource of immense interest to both academia and pharma and appropriate strategic partnerships could be launched.

## Theme 4: Living with and beyond

### Patient and public comments

Of a number of discrete priorities that emerged, one participant questioned whether more could be done to educate people who continue to live with the disease and improve their standard of living through the assistance of specialists—

ʻCould the education (and quality of life) of patients living with & beyond lung cancer be improved with ongoing support, not just from clinical oncologists ……...ʼ

Individuals queried whether exercise should be promoted following diagnosis, and treatment, with others highlighting the importance of exercise on improving lung function—

‘*Keeping active is evidenced to improve lung function…*

*why is more not suggested to patients pre and post their treatment to remain as active as possible and increase activity to develop their lung function?*’.

### Evidence

Firstly there was the confirmation of the importance of theme 1 and an emphasis that educating society in general and healthcare professionals in particular needs to continue beyond the diagnostic phase of the patient’s journey, with the LCINS population having different needs and treatment options in comparison to those with smoking-related lung cancer.

While recognising that attitudes are changing and over the last 10 years there has been an improvement in the fatalistic view of a lung cancer diagnosis amongst oncologists, among the general public there remains a stigma associated with lung cancer diagnosis [99]. Most individuals agree with the statement ‘*lung cancer patients are at least partially to blame for their illness*’, with half of people living with lung cancer experiencing blame from strangers and acquaintances and a quarter of individuals feeling less supported by family and friends [100]. Changing cultural and societal approaches to lung cancer stigma is challenging. Paradoxically the concerted efforts to improve awareness to stop smoking may have increased the stigma surrounding a lung cancer diagnosis impacting on support networks for these patients, further alienating those with LCINS who do not ‘fit the profile’ of a lung cancer patient.

There is little evidence about the specific needs and experiences of never smokers and therefore we have a limited understanding about whether they have different needs to smokers or how best to support this group. The evidence relating to stigma and the unexpected diagnosis of lung cancer may impact on adjustment to a cancer diagnosis and lead to specific information needs. There is some suggestion that health-related quality of life (HRQoL) is better in those who quit smoking or are never smokers, with Rowland [101] suggesting that needs may be different and this warrants further investigation.

There is limited long-term data on survival of LCINS, some studies suggest that this population has a better outcome when compared to the smoking-related lung cancer population [12, 31, 102, 103]. This is despite presentation with vague symptoms and a lack of awareness of the possibility of a lung cancer diagnosis that means LCINS tend to present late with more advanced disease. While studies suggest around 66% presented with stage IV disease [48, 104] they do not show any no clear difference when compared to the stage distribution seen in the smoking population [12].

### Treatment

Treatment for LCINS should follow the same principles as those that guide the management of smoking-related lung cancer and all should be given the opportunity to participate in research studies. UK lung cancer trial recruitment figures show a 10-fold variation across the country [105]. This sits alongside the geographical variations seen in service, issues which need addressing for both the smoking and never smoking population. For those presenting with early-stage disease the focus needs to be on potentially curative treatment with surgery and/or radiotherapy. For the LCINS population knowledge of mutation and biomarker status is of crucial importance in guiding adjuvant systemic treatment [106–108].

Systemic therapy is the cornerstone of treatment for those presenting with advanced metastatic disease [109, 110]. It is within the systemic treatment setting that the approach to treatment is likely to differ most from that offered to the smoking population. In general, the LCINS population are younger with fewer co-morbidities at the time of diagnosis and better able to cope with the increased toxicity associated with multi-modality treatments [111, 112]. This population will also have a significantly higher levels of druggable driver mutations [6] for whom a sequential approach to systemic treatment may be more appropriate than using a combination treatment in the first line setting. Hence, it is particularly important that we move on from platinum-based chemotherapy to a more individualised approach to systemic treatment based on the results of rapid genomic testing at the time of diagnosis.

As discussed in Theme 3 there is a clear priority to develop new treatment for LCINS. However, it is equally important that we get a better understanding of the sequencing of those currently available (targeted, immunotherapy, chemotherapy) to limit/delay the development of tumour resistance [110, 113]. For example, the standard treatment approach for drugs targeted at mutation drivers is treat to progression where the resistant clones predominate and drive the course of the disease. An adaptive approach with drug ‘holidays’ or sequencing of drugs targeting different pathways to delay the emergence of the resistant clone have been proposed and studies evaluating this approach could be developed by investigator led groups.

The role of radiotherapy for the advanced LCINS population is also poorly understood. While palliative radiotherapy is widely used in stage IV NSCLC and has proven effective at controlling symptoms and may improve survival, the evidence comes from studies conducted in the last century. Since then, there have been major improvements in systemic therapies and it is increasingly recognised that in modern practice, palliative radiotherapy is still offered to many patients with advanced lung cancer, based on increasingly dated evidence. This is particularly true for the LCINS population who were barely represented in previous clinical trials and need studies to test the timing, dose and fractionation of radiotherapy before, during or after modern systemic therapies.

Over the past two decades, there have been significant advances in radiotherapy planning and delivery, allowing higher dose treatments to be given more accurately, improving responses while reducing toxicity and there is the opportunity to develop specific arms in some of the current radiotherapy studies for palliative thoracic radiotherapy (TOURIST) [114], oligometastatic (SARON) (ClinicalTrials.gov identifier NCT02417662) or oligo-progressive (HALT) disease (ClinicalTrials.gov identifier NCT03256981).

### Recommendations

There remains a stigma associated with a diagnosis of lung cancer, due to the well-established association with smoking. By addressing theme 1 and educating people of the risk factors associated with lung cancer among those who have never smoked, we may not only reduce this stigma, but also help to identify and diagnose LCINS earlier, aiding survival rates.

- As most of the research is around ever smokers, more research is needed to explore the views, needs, experiences and HRQoL of never smokers diagnosed with lung cancer. This will help identify differences in the support, informational and care needs of never smokers to develop or target evidence-based psychosocial interventions.
- Studies are required to identify the optimal methods for managing advanced lung cancer for the LCINS population focusing on patient support, adapting and integrating available treatment modalities. By doing so, we can help never smokers living with lung cancer by providing bespoke support if this differs from that of ever smokers.

## Conclusions

We present a research strategy to address the unmet needs of patients diagnosed with lung cancer who have never smoked. Key recommendations-

1. Raising awareness of LCINS in the general population and with healthcare professionals, with a specific focus in primary care.
2. Develop a national registry for the LCINS population to further our understanding of the patient journey including barriers to diagnosis. The clinical and radiomic data collected should aim at evaluating inherited and environmental risks in the LCINS population to identify higher risk groups to prioritise for screening and develop approaches to improve disease prevention and earlier diagnosis. We also recommend establishing prospective cohorts with associated biological sample collection. The aim is to develop a resource to better characterise disease biology/genetic mutational changes aiding the development of more precise risk prediction and novel treatments.
3. Clinical trials are required to identify the optimal methods for managing advanced lung cancer in the never smoking patient population focusing on patient support, adapting and integrating available treatment modalities.

The NCRI is committed to supporting research to improve LCINS outcomes, maintaining a focus on LCINS as a key priority. This will entail an ongoing dialogue with patient advocates as well as strategic collaborations within academia and with biotech/pharma sectors.

## Data Availability

Data is available upon request from the corresponding author.
